# Hyperbaric oxygen therapy in China

**DOI:** 10.1186/s13618-015-0024-4

**Published:** 2015-02-18

**Authors:** Ling Yan, Ting Liang, Oumei Cheng

**Affiliations:** Department of Neurology, The First Affiliated Hospital, Chongqing Medical University, Chongqing, 400016 China

**Keywords:** Hyperbaric oxygen therapy, China, Indications and contraindications, Clinical and basic research

## Abstract

Hyperbaric oxygen therapy (HBOT) is defined as a treatment in which a patient intermittently breathes 100% oxygen while the treatment chamber is pressurized to a pressure greater than sea level (1.0 atmosphere absolute, ATA). In China, for nearly 50 years, HBOT has been used as a primary or adjuvant therapy to treat a variety of diseases. This article mainly reviewed the indications and contraindications of HBOT, as well as the status of clinical and experimental HBOT research in China. At the same time, there is a brief introduction of hyperbaric oxygen preconditioning (HBO-PC) in China.

## Background

In 1887, Valenzuela successfully pioneered the application of 2.0 Mpa of pure oxygen to treat clinical disease [1], the use of hyperbaric oxygen therapy (HBOT) for clinical practice has a history of more than 100 years from then on. The hyperbaric oxygen chamber was first used in China by the Fujian Medical University Union Hospital in 1964 [2]. Since then, HBOT has experienced nearly 50 years of rapid development in China. The basic principle behind HBOT is to increase the amount of oxygen dissolved in the blood by administering it at a pressure greater than sea level (1.0 atmosphere absolute, ATA). In this way, the pressure gradient will distribute O_2_ throughout the body and maintain tissue in a hyper-oxygenated state [3]. Currently, there are more than 5000 hyperbaric oxygen chambers in China [4]. This number is the highest in the world, and significantly contributes to the amount of global HBOT research [4]. In China, HBOT has been adapted to treat a wide variety of diseases. This paper reviewed the current clinical applications of HBOT (including indications and contraindications) and both clinical and basic HBOT research in China in recent years to provide theoretical evidence for a rational clinical use of HBOT.

## HBOT in clinical practice

HBOT use in China began in the 1960s and developed rapidly after political reform and opening-up. As a result of this expansion of the practice of HBOT, a platform was urgently needed to develop standards of practice. To address this concern, the Chinese Medical Association (CMA), which was established in 1915 as the institution for the development of medical science and technology in China, organized a branch for hyperbaric oxygen medicine in 1992. Since then, the branch of hyperbaric oxygen medicine of the CMA has held annual academic conferences. These conferences provide a platform for academic exchange for the majority of HBO therapists in China. The internet communication platform, China Hyperbaric Oxygen (CHO) was created in 2000 by QingLe Liu, the director of the Second Military Medical University Medical Center of Hyperbaric Oxygen. Through those platforms, HBOT has gradually moved toward standardization. HBOT is commonly used on patients with ischemic hypoxic damage. With the wide clinical application of hyperbaric oxygen therapy, the Chinese Professional Committee of Hyperbaric Oxygen drafted the indication and contraindication standards of practice in 1982 in an effort to reduce the number of patients experiencing toxic side effects of HBOT. The CMA revised the indication and contraindication standards in 2004 and 2013; the 2004 version is widely used in clinical practice, followed by the indications and contraindications of 2004.

### The indications of HBOT

Indications refer to the scope and standards for the suitable use of HBOT. A few countries other than China have developed their own standards for HBOT. In the United States, in 1989, the Undersea and Hyperbaric Medical Society (UHMS) formulated indications for HBOT use that include 13 diseases [5]. In 2014, the number of indications increased to 17 (Table 1). In 2004, the European Committee for Hyperbaric Medicine (ECHM) divided their recommended indications into 4 categories: 8 highly recommended indications, 10 recommended indications, 9 controversial indications,and 13 other indications that include 40 other diseases [6]. Compared to the United States and Europe, the number of hyperbaric oxygen indications approved in China is relatively high. The indications of HBOT were initially released in China in 1982. With the practice and recognition of HBOT, the CMA revised the recommended indications in 2004 [4] to include 12 emergency indications and 48 non-emergency indications.

**Table 1:** **HBO Indications of UHMS**

Air or gas embolism;	Diabetically derived illness, such as diabetic foot, diabetic retinopathy, diabetic nephropathy;
Carbon monoxide poisoning;	Exceptional blood loss (anemia);
Carbon monoxide poisoning complicated by cyanide poisoning;	Idiopathic sudden sensorineural hearing loss;
Central retinal artery occlusion;	Necrotizing soft tissue infections (necrotizing fasciitis);
Clostridal myositis and myonecrosis (gas gangrene);	Intracranial abscess;
Crush injury, compartment syndrome, and other acute traumatic ischemias;	Osteomyelitis (refractory);
Decompression sickness;	Delayed radiation injury (soft tissue and bony necrosis);
Enhancement of healing in selected problem wounds;	Skin grafts and flaps (compromised);
Thermal burns.	

Emergency indications are diseases where HBOT should be administered as soon as possible. The following are emergency indications: (1) acute carbon monoxide poisoning and other harmful gas poisoning; (2) gas gangrene, tetanus and other anaerobic bacteria infections; (3) decompression sickness; (4) air embolism syndrome; (5) after cardiopulmonary resuscitation (CPR) due to a variety of risks for acute brain dysfunction; (6) aid in the treatment of shock; (7) brain edema; (8) pulmonary edema (except cardiac pulmonary edema); (9) crush syndrome; (10) limb (finger, toe) and the blood supply after skin transplantation; (11) drug and chemical poisoning;(12) acute ischemia anoxic encephalopathy.

Additionally, the following non-emergency indications are approved for use: (1) carbon monoxide poisoning or other toxic encephalopathy; (2) sudden deafness; (3) ischemic cerebrovascular disease (cerebral arteriosclerosis, transient ischemic attack, cerebral thrombosis, cerebral infarction); (4) craniocerebral injury (concussion, cerebral contusion of intracranial hematoma removal surgery, brain stem injury); (5) cerebral hemorrhage recovery; (6) poor healing fractures; (7) central serous retinal inflammation; (8) vegetative state; (9) plateau adaptation insufficiency syndrome; (10) peripheral nerve injury; (11) intracranial benign tumor surgery; (12) periodontal disease; (13) viral encephalitis; (14) facial paralysis; (15) osteomyelitis; (16) aseptic osteonecrosis; (17) cerebral palsy; (18) fetal developmental delays; (19) diabetes and diabetic foot; (20) coronary atherosclerotic heart disease (angina and myocardial infarction); (21) rapidity arrhythmia (atrial fibrillation, premature beat, tachycardia); (22) myocarditis; (23) peripheral vascular disease, vasculitis, e.g., Raynaud’s, deep vein thrombosis, etc.; (24) vertigo; (25) chronic skin ulcer (arterial blood supply obstacles, venous congestion, bedsore); (26) spinal cord injury; (27) peptic ulcer; (28) ulcerative colitis; (29) infectious hepatitis (use the special chamber of infectious disease); (30) burns; (31) frostbite; (32) plastic surgery; (33) skin grafting; (34) sports injuries; (35) radioactive damage (bone and soft tissue, cystitis, etc.); (36) malignant tumors (with radiotherapy or chemotherapy); (37) otic nerve injury; (38) fatigue syndrome; (39) angioneurotic headache; (40) pustular; (41)psoriasis; (42) pityriasisrosea; (43) multiple sclerosis; (44) acute Guillain-Barre syndrome; (45) recurrent oral ulcer; (46) paralytic ileus; (47) bronchial asthma; and (48) acute respiratory distress syndrome.

The current HBOT indications and contraindications were released at the 22nd academic meeting held in Qingdao in 2013 and approved on the 1st of November 2013. The new indications include diseases that were directly or indirectly caused by hypoxia and/or ischemia or a series of conditions that are related to hypoxia and/or ischemia in the evolution of the disease process. In comparison to the 2004 edition, the new indications broaden the use of HBOT.

### The contraindications of HBOT

Contraindications refer to the diseases or predicaments where the use of HBOT is inappropriate, possibly leading to adverse consequences, including body injury and even death. The CMA published the contraindications of hyperbaric oxygen treatment in 2004, which includes 4 absolute contraindications and 10 relative contraindications.

Absolute contraindications are those where HBOT is prohibited if the patient is accompanied with following: (1) untreated pneumothorax, untreated pneumomediastinum; (2) pulmonary bulla; (3) active hemorrhage and hemorrhagic disease; or (4) the formation of tuberculous cavity and hemoptysis.

Relative contraindications refer to the conditions where the use of HBOT in patients is cautioned and may possibly lead to side effects that increase discomfort or complications. Thus, HBOT should be used with caution if a patient has one of the following conditions: (1) severe upper respiratory tract infection; (2) severe emphysema; (3) bronchiectasis disease; (4) sinus infection; (5) aII degree atrioventricular block; (6) high blood pressure (>160/100 mmHg); (7) bradycardia (<50 times/min); (8) untreated malignant tumor; (9) retinal detachment; and (10) the early stage of pregnancy (3 months).

In 2013, new contraindications for HBOT were released by CMA. The new contraindications include absolute contraindications and relative contraindications. The only absolute contraindication is tension pneumothorax without treatment. The relative contraindications are as follows: (1) intraventricular external drainage; (2) fracture of the skull base with cerebrospinal fluid leakage; (3) birth weight < 2000 g in premature and low birth weight infants; (4) serious infection of the upper respiratory tract; (5) high blood pressure (SBP > 180 mmHg, DBP > 110 mmHg); and (6) patients with chronic obstructive pulmonary disease with CO_2_ retention. Compared to the previous contraindications, the current contraindications are less strict. For example, regarding blood pressure, the 2004 standard is > 160/100 mmHg, but the new standard is > 180/110 mmHg. Still, adverse effects can occur even when following stricter HBOT contraindications.

## Clinical and basic research of HBOT in China

**Figure 1:**
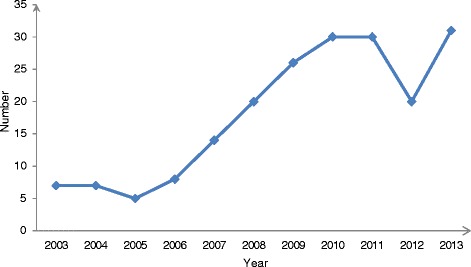
**The number of published articles on hyperbaric oxygen in China.**

HBOT has been widely adopted since its introduction in China. With the wide application of HBOT in clinical settings, a substantial amount of research relevant to HBOT has been published. According to the Chinese National Knowledge Infrastructure (CNKI), there are 13,357 articles related to hyperbaric oxygen (before Aug 2013). Using “hyperbaric oxygen” and “China” as key words, 218 articles appeared in PubMed (before Aug, 2013). This amount of hyperbaric oxygen research suggests that HBOT in China is still in the development stage. The amount of published HBOT research has been increasing every year for nearly a decade (2003–2013). However in 2012, there was a decline in HBOT research (Figure 1).

Here, we summarized HBOT research in China using original research articles found in PubMed that focus on both clinical and basic research.

### Clinical research

A lot of clinical research on HBOT has been published recently. Within the last ten years (2004–2013), 9 randomized controlled trials (RCTs) have been published. The patients used in those studies had a variety of disorders, including neurologic, otolaryngologic, stomatologic, dermatologic, and urologic disorders. The cohorts for those trials ranged in size from 24 to 236. The Jadad – Bechara scale is often used to assess the quality of a clinical trial study. The Jadad scale (Table 2), which was developed by Columbia doctor, Alejandro Jadad – Bechara, is sometimes referred to as the Jadad score or the Oxford scoring system and is the most widely used rating scale for clinical trial research [7]. According to the Jadad scale, a score between 1 and 3 indicates a low quality study, where as a score between 4 and 7 indicates a high quality study [8]. The 9 RCTs found within the last ten years are summarized and graded for their quality in Table 3 [9-15].

**Table 2:** **The Jadad score**

Generation of allocation sequence	0 no double-blinding
2 computer-generated random numbers	Description of withdrawals and drop-outs
1 not described	1 numbers and reasons are described
Allocation concealment	0 numbers and reasons are not described
3 central randomization	Efficacy of randomization
2 sealed envelopes or similar	2 pre-treatment variables in tabular from
1 not described or inadequate	1 balance of pre-treatment variables
Investigator blindness	1 balance of pre-treatment variables mentioned but not in tabular form
2 identical placebo tables or similar	0 no information report
1 inadequate or not described

**Table 3:** **HBOT RCTs in 2004–2013 and the quality score**

**The study**	**Published time**	**Sample size**	**Quality score**	**Study population**	**The effect**
Cao H	2013 Jun	30/30	2	Depression in the convalescent stage following cerebral hemorrhage	effective
Chen TL	2012 Oct	30/30	2	Aggressive periodontitis and subgingival anaerobes	effective
Peng Z	2012 Nov	68	2	Patients with herpes zoster	effective
Tang XP	2011 Nov	60/60	2	Postoperative patients with intracranial aneurysm	effective
Shao Y	2012 Mar	18/18	2	Radiation-induced hemorrhagic cystitis	effective
Tang X	2011 Mar	116/116	2	Meningiomas with conspicuous peritumoral brain edema	effective
Jiang W	2011 Jan	48/35	2	Late healed wounds after pharyngeal and laryngeal surgery	effective
Liu Y	2010 Oct	60/60	1	Sudden deafness patients	effective
Yuan JB	2011 Sep	12/12	1	Erectile function after posterior urethral reconstruction	maybe effective

The HBO RCTs in China were generally of low quality (scores ranged from 1–2). Thus, the curative effect of HBOT cannot be determined from these studies. For a more rigorous test of the curative effects of HBOT, future experiments should sea stricter research design that includes a randomized, double blind, and multicenter approach, as well as a large sample.

### Basic research

Although the therapeutic effect of HBOT has been confirmed in the clinic, the mechanism is not fully understood. The principle strategy of HBOT is to increase the oxygen content of the blood, improve blood oxygen partial pressure, improveblood oxygen dispersion and increase the tissue oxygen “effective diffusion distance” while constricting blood vessels and promoting the establishment of collateral circulation. HBOT can affect many physiological processes. In different disease states, HBOT is associated with decreased apoptotic cell death, reduced inflammation, a balance of oxygen free radicals, and the activation of stem cells and other mechanisms. I will mainly describe the next four mechanisms, and most of the related diseases are mainly involved in nervous system.

#### The inhibition of cell apoptosis

HBOT inhibits cell apoptosis by regulating apoptosis-related proteins in a variety of pathological models. HBOT can reduce the secretion of caspase-3, which is critical for apoptosis induced by proteases [16] in different rat models of ischemia. These models include brain ischemia-hypoxia in neonatal rats [17] and cerebral ischemia-reperfusion injury in adult rats [18]. In addition, similar anti-apoptotic effects have been reported in models of myocardial infarction [19]. B-cell lymphoma 2 (Bcl-2) is an inhibitor of the apoptosis protein, Bax, which is a Bcl-2-related protein that pro-apoptotic. The ratio of Bcl-2/Bax is up regulated by HBOT and inhibits cell apoptosis in a rat model of myocardial infarction [19]. In addition, Thioredoxin Reductase(TrxR), an antioxidant, also has an antiapoptotic effect that can involve HBOT [20]. For example, HBOT is beneficial for the improvement of anxiety-like behavior and cognitive impairments in stressed rats; this effect might be associated with the inhibition of neuronal apoptosis via up regulation of TrxR [21].

#### The reduction of inflammation

HBOT can reduce inflammation by reducing the release of inflammatory mediators. Such inflammatory mediators include the lymphokine (IL) family and tumor necrosis factor (TNF). For example, HBOT attenuated inflammation via the reduction ofIL-1, IL-6, IL-8, and IL-10expression in rat models of ischemia or injury [22-24]. Additionally, HBOT reduced TNF-α in a model of testicular ischemia-reperfusion injury in rats [25]. Besides, HBOT can also reduce inflammation by reducing the incidence of membrane cofactor protein-1(MCP-1), keratinocyte-derived chemokine (KC), and IFN-gamma-inducible protein 10(IP-10) [26]. In addition, the anti-inflammatory effect of HBOT may be related to the inhibition of specific signaling pathways. HBOT may mitigate secondary injury to the spinal cord(SC) by inhibiting inflammatory responses induced by the TLR2/NF-кB and the iNOS mRNA-iNOS-NO signaling pathways, thereby promoting functional recovery in spinal cord injury in rats [27,28].

#### The balance of oxygen free radicals

Oxygen free radicals are a product of metabolic processes of the body. Because oxygen free radicals generally exist in the body, their production and scavenging are normally in a dynamic equilibrium. The appropriate levels of oxygen free radicals can facilitate tissue metabolism and cell detoxification, but excessive oxygen free radicals will damage the body [4]. In theory, HBO can increase oxygen free radicals. HBOT initiated soon after acute transient cerebral ischemia in rats increases mitochondrial free radical levels but also increases the activity of superoxide dismutase (SOD), which is important for scavenging free radicals. Thus, the net effect is an increase in oxygen and a reduction in oxygen free radicals, which then protects the body [29,30].

#### Activation of neural stem cells

A stem cell is a type of undifferentiated immature cell that has the potential for proliferation, migration and differentiation into all types of tissue cells; this is beneficial for the recovery of damaged tissue. Neurogenesis plays an important role in the recovery of neurological function. HBOT activation of stem cells may represent a viable new research direction. Wang XL found that in newborn rats with hypoxic ischemic brain damage (HIBD), HBOT could promote migration and differentiation of endogenous neural stem cells (NSCs) in the subventricular zone (SVZ) into cerebral cortex neurons [31], but its mechanism still need further research. HBOT can promote neurogenesis in the piriform cortex(Pir) of adult rats with vascular dementia(VD) [32]. HBOT has a different effect at different pressures and different exposure times. HBOT at 2 ATA for 60 minutes produces the highest levels of differentiation of NSCs into neurons in cerebral cortices of newborn rats [33]. Current research on the effects of HBOT on neurogenesis mainly involves NSC activation, but research using other types of stem cells remains to be explored in the future.

### Hyperbaric oxygen preconditioning (HBO-PC)

HBO pretreatment can have a protective effect that alleviates the secondary damage after some stimulation; this is known as hyperbaric oxygen preconditioning (HBO-PC). In a clinical trial of forty-nine elective on-pump or off-pump coronary artery bypass graft surgery patients, Li Y found that HBO-PC improved the outcome of patients undergoing on-pump coronary artery bypass graft surgery. The on-pump group had a reduced length of stay in the intensive care unit and a decreased use of inotropic drugs [34]. Although the mechanism of precondition is not fully understood, animal research suggests that HBOT may mitigate surgery-induced cognitive impairment [35] and reduce ischemia-reperfusion (IR) injury of the rat brain [36]. HBO-PC can reduce the number of apoptotic cells and promote nerve functional recovery [37,38]. The protection mechanism may involve reduction of systemic and hippocampal proinflammatory cytokines, reduction of COX-2 (an inflammatory medium) [39], suppression of caspase-3 activity [35], or up regulation of HSP32 expression (Heat Shock Protein 32, a protein that inhibits normal cell death) [40].

## Summary

HBOT in China has a wide range of indications, involving nearly every system of the human body. However, contraindications are relatively limited. Although the use of HBOT in China for the clinical treatment of various diseases has been widely studied, the quality of these clinic trials is generally low due to a small sample size and high heterogeneity between studies. Research with a stricter design that includes, random trials, blind experimentation, multiple centers, and large samples are needed for further confirmation. The mechanism of HBOT has been examined in animal model studies, which mainly report improvement of microcirculation, decrease in apoptotic cells, reduction of inflammation, adjustment of the balance of oxygen free radicals, and the activation of stem cells. Still, the mechanism of HBOT in the human body is not fully understood. Further study using advanced non-invasive techniques, such as Positron Emission Tomography Computed Tomography (PET-CT) and functional Magnetic Resonance Imaging (fMRI) may help discover additional mechanisms. HBO-PC can reduce injury to the body in some cases, and its prospects for clinical application as well as the in-depth study of its mechanism will be of broad interest.

The number of published articles on hyperbaric oxygen is increasing year by year, but in 2012, there was a decline (From PubMed).

The clinical RCTs of HBO in China are generally of low quality (quality score ranged from 1–2). Definitive curative effects of HBOT cannot be concluded from these studies.
